# A 4D IMRT planning method using deformable image registration to improve normal tissue sparing with contemporary delivery techniques

**DOI:** 10.1186/1748-717X-6-83

**Published:** 2011-07-19

**Authors:** Xiaoqiang Li, Xiaochun Wang, Yupeng Li, Xiaodong Zhang

**Affiliations:** 1 Department of Radiation Physics, The University of Texas, MD Anderson Cancer Center, Houston, Texas 77030, USA

**Keywords:** 4D CT, IMRT, treatment planning, respiratory motion, deform

## Abstract

We propose a planning method to design true 4-dimensional (4D) intensity-modulated radiotherapy (IMRT) plans, called the t4Dplan method, in which the planning target volume (PTV) of the individual phases of the 4D computed tomography (CT) and the conventional PTV receive non-uniform doses but the cumulative dose to the PTV of each phase, computed using deformable image registration (DIR), are uniform. The non-uniform dose prescription for the conventional PTV was obtained by solving linear equations that required motion-convolved 4D dose to be uniform to the PTV for the end-exhalation phase (PTV50) and by constraining maximum inhomogeneity to 20%. A plug-in code to the treatment planning system was developed to perform the IMRT optimization based on this non-uniform PTV dose prescription. The 4D dose was obtained by summing the mapped doses from individual phases of the 4D CT using DIR. This 4D dose distribution was compared with that of the internal target volume (ITV) method. The robustness of the 4D plans over the course of radiotherapy was evaluated by computing the 4D dose distributions on repeat 4D CT datasets. Three patients with lung tumors were selected to demonstrate the advantages of the t4Dplan method compared with the commonly used ITV method. The 4D dose distribution using the t4Dplan method resulted in greater normal tissue sparing (such as lung, stomach, liver and heart) than did plans designed using the ITV method. The dose volume histograms of cumulative 4D doses to the PTV50, clinical target volume, lung, spinal cord, liver, and heart on the 4D repeat CTs for the two patients were similar to those for the 4D dose at the time of original planning.

## 1. Introduction

Implementations of four-dimensional (4D) radiotherapy based on 4D computed tomography (CT) datasets have been described by Rietzel *et al *[1] and Keall [2]. In 4D radiotherapy, the treatment plan is designed on each 4D CT image set (i.e., 4D treatment planning), and radiation is delivered throughout the patient's breathing cycle (i.e., 4D treatment delivery), which ensures adequate coverage of the tumor target without increasing the treated volume. Because 4D treatment planning accounts for temporal changes in anatomy, 4D radiotherapy holds promise as the optimal method for treating patients. However, 4D radiotherapy currently requires 4D treatment delivery, which necessitates sophisticated device(s) to synchronize the treatment delivery with the patient's respiration. Most centers have the ability to acquire 4D CT images, but they do not have the ability to perform 4D radiation delivery. Instead, 4D CT images are primarily used to define the internal target volume (ITV), which is essentially the envelope needed to enclose the target as it moves throughout the breathing cycle. 4D CT [3-9] provides a more accurate tumor volume definition since it limits motion artifacts during CT acquisition, displays the anatomically correct shape and size of the tumor, and demonstrates respiration-induced motion of the tumor and organs at risk. Previous studies using 4D CT datasets have mostly been focused on dosimetric verification to determine if dose distribution planned on one or part of the 4D CT datasets is adequate to estimate the cumulative dose from all 4D CT datasets [1,10]. Few studies have investigated whether the information on anatomic motion provided by 4D CT can be used to design treatment plans that confer the advantages of 4D treatment delivery without requiring additional equipment.

**Figure 1:**
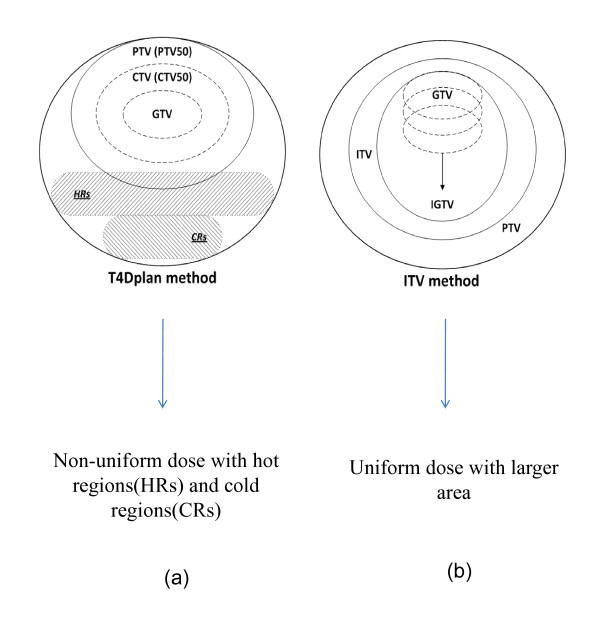
**The difference between (a) t4Dplan method and (b) traditional ITV method**. The planning target volume (PTV) in the ITV method is the target volume used to plan and treat. In the t4Dplan method, the PTV50 plus the hot regions (HRs) are the target volume used to plan and treat. The cold regions (CRs) in the t4Dplan method represent the reduced treated volume relative to that in plans from the ITV method. CTV represents the clinical target volume; GTV represents gross tumor volume; IGTV represents internal gross tumor volume.

In this paper, we describe an effective and practical 4D treatment planning method, which we refer to true 4D planning (t4Dplan) method, for intensity-modulated radiotherapy (IMRT) using 4D CT datasets to maximize critical structure sparing. In traditional treatment planning, the prescribed dose is planned to be distributed uniformly to the target while minimal dose is delivered to the surrounding normal structures on the planning CT under the assumption that the planning CT truly represents the patient anatomy that will be present during treatment. In our t4Dplan method, however, planning deliberately creates non-uniform dose distribution in the target (i.e., it creates hot regions along the target's direction of motion on the planning CT) to achieve a uniform dose distribution in the target and minimal dose to the surrounding normal structures on the final 4D dose distribution. The difference between the t4Dplan method and the traditional ITV method is illustrated in figure 1. The t4Dplan method does not require 4D treatment delivery and is solely dependent on the 4D datasets acquired during the planning process. Compared to some other techniques such as respiratory gating [11], breath hold [12,13] and dynamic MLC tumor tracking [14-16], the t4Dplan method is easier to implement in the clinic because it uses the current treatment planning and delivery systems.

## 2. Materials and methods

### 2.1. t4Dplan

The t4Dplan method, which uses 4D CT datasets, designs treatment plans as follows:

1. A reference CT dataset is selected from all the 4D CT datasets. Usually, an end-of-exhalation phase CT (i.e., the 50% phase [T50]) is selected as the reference CT dataset [17] since patients spend more time at the end of exhalation [18].

2. The target volume (TV) is outlined based on the reference CT.

3. The motion TV (MTV) is outlined on the reference CT as the combined volume of the target at all phases of the 4D CT datasets (i.e., the MTV is an envelope enclosing the target as it moves throughout the breathing cycle).

The t4Dplan method calculates a deliverable non-uniform dose distribution (i.e., the apparent dose distribution [AppD]) to the MTV. The final 4D dose distribution is determined by recalculating the t4Dplan on each phase of the 4D CT dataset and creating a time-averaged cumulative dose distribution based on deformable image registration (DIR).

For each voxel on the reference CT, the corresponding voxel on another phase of the CT dataset can be derived through DIR by transforming the source image (i.e., the reference CT) to the target image (i.e., another phase of the CT dataset), such that(1)

where  is the position vector for the *i*th voxel on the reference CT, *T*^*j,ref *^represents the transform matrix from the reference CT to the *j*th phase of the CT dataset, and  is the position vector for the corresponding voxel on the *j*th phase of the CT dataset for the *i*th voxel on the reference CT.

In the current study, to derive the non-uniform dose, we first assumed that the dose on each phase of the 4D CT was approximately the same as the AppD on the reference CT. This approximation assumes the internal movement of anatomy will not impact the dose distribution and is a good approximation for photon dose calculation. It should be noted that this approximation is only used in the derivation of a non-uniform dose prescription. For the final designed plan, we used the exact 4D dose calculation without this approximation. The 4D dose for each voxel on the reference CT can be approximated as the time-averaged cumulative dose of the corresponding voxel on all phases in the CT dataset, such that(2)

where *K *represents the number of phases of the CT datasets,  is the 4D dose for the *i*th voxel on the reference CT, and  is the AppD for the corresponding voxel on the *j*th phase of the dataset.

**Figure 2:**
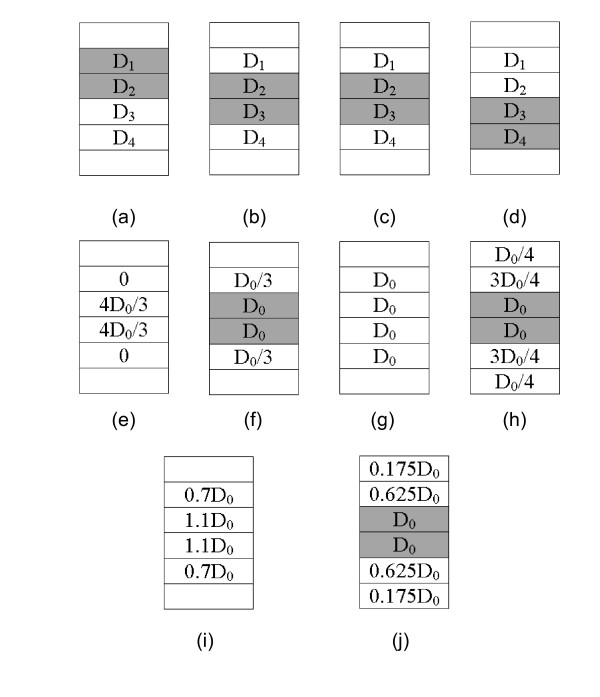
**A phantom with tumor volume (shadow area) moving only in superior-inferior direction was illustrated to show how the non-uniform dose distribution was derived**. The moving circle was divided into 4 phases (a), (b), (c), (d). The non-uniform prescribed dose distribution derived by the t4Dplan method was shown on (e) and its corresponding 4D dose was shown on (f) by summing the dose for 4 phases evaluated on (b). The prescribed dose for ITV approach (g) and the 4D dose (f). The non-uniform dose distribution acquired for t4Dplan with the total variation regulation from formula (5) was shown in (i) and its corresponding 4D dose (j).

Assuming the MTV and TV on the reference CT have *n *and *m *(*n *>*m*) voxels, respectively, and the AppD values for the *n *voxels of the MTV are *D*_*1*_, *D*_*2*_, ..., *D*_*n*_, the 4D dose distribution for the TV with *m *voxels can be determined using the following linear equations derived from equation (2):(3)

where , or *D*_*n*_, are the unknown parameters, and *D*_*0 *_is the uniform dose prescribed to the TV (i.e., the final 4D dose distribution on the TV). Here, we have *n *unknown parameters (i.e., *D*_*1*_, *D*_*2*_, ..., *D*_*n*_) that need to be derived from *m *equations, with *m *<*n*. The solution for equation group (3) is underdetermined. In order to clarify the idea how the linear equations are constructed and the non-uniform dose distribution is derived more clearly, we used a simple phantom (shown in figure 2) to illustrate. This phantom shown in figure 2(a, b, c, d) had 4 phases, the MTV had 4 voxels (voxel 1-4), the TV had 2 voxels (shadow area). So the linear equations were constructed by 4D dose convolution of each TV voxel as follows:(4)

Since the linear equations had 4 unknown parameters and only 2 equations, it was undetermined. One of the solutions can be acquired by applying an extra constraint, which implied the smallest margin (*D*_1 _= *D*_4 _= 0), and was shown figure 2(e). The corresponding 4D dose referenced on the second phase (figure 2(b)) was calculated and shown on figure 2(f). Compared to the ITV approach, distributing uniform prescribed dose to all the voxels (figure 2(g)), resulting 4D dose (figure 2(h)) when accumulating for all phases, the non-uniform dose distribution clearly decreased the margin, spared the critical organ while maintaining the same target coverage (figure 2(f) vs figure 2(h)).

The non-uniform dose as shown is figure 2(e) was an ideal prescribed dose, assuming the dose can be delivered for this pattern. In reality, when delivering a high dose to a specific voxel, it is impractical to achieve a very low dose in the nearby voxels due to the dose fall-off gradient. For this reason, one possible AppD can be acquired by minimizing the following objective function with a total-variation regulation [19]:(5)(6)

where *n *is the total number voxels of MTV on the reference CT dataset. The parameter *λ *is the importance factor to penalize the second term of (5) which calculates the sum of absolute derivatives. The penalty tends to have zero derivatives and smoothes the voxels' prescribed doses for easy delivery. The formula (6) constrains the maximum inhomogeneity to be 20%. The reason is that when we create the apparent plan with designed hot region, we want the apparent plan not to be too hot. In our routine clinical practice, our clinician sometimes also accepts the plan 20% hot in the PTV, therefore, the plan can be readily used in the routine clinical practice. For the phantom in figure 2, the solution to minimize equation (5) was shown in figure 2(i). Compared to the ideal solution (figure 2(e)), the solution with the total-variation regulation blurred the non-uniform dose and created a more natural dose fall-off, which became practical for deliverable optimization. The corresponding non-uniform 4D dose, as shown in figure 2(j), still had enough dose coverage to the target and more normal tissue sparing than that of the ITV approach (figure 2(j) vs figure 2(h)).

The derived AppD for the MTV served as the non-uniform dose prescription for the MTV. The deliverable AppD can be obtained using an IMRT optimization system. The voxel-based optimization function was used to achieve the AppD for the MTV on reference CT, such that(7)

In other words, the derived AppD for the MTV becomes the optimization objective for the MTV. The Pinnacle treatment planning system (version 8.1x, Philips Medical Systems, Milpitas, CA) was used as the platform for IMRT optimization. The in-house-developed plug-in module, which optimizes the dose distribution to achieve the derived AppD for the MTV using equation (7), cooperates with the Pinnacle IMRT optimization system to achieve the final deliverable AppD on the reference CT, which results in a uniform dose to the TV and minimal dose to the surrounding critical structures for 4D dose distribution. In our implementation, only the objective function (7) was added to the Pinnacle inverse planning module as a plug-in. The conventional objectives that are not voxelized can still be used to control normal tissue sparing and target coverage. From the treatment planner's point of view, our method is an enhancement of the current planning method. This implementation makes our method readily available for use in routine clinical practice.

Once treatment optimization based on the AppD was obtained, the dose on each 4D CT was recalculated and the 4D dose distribution obtained by using DIR.

### 2.2. Evaluation of t4Dplan method

Three patients with tumor located in the middle lobe of the right lung (patient 1), near the diaphragm of the left lung (patient 2) and near the diaphragm of the right lung (patient 3) respectively were selected for our evaluation of the t4Dplan method. The characteristics of the three patients were listed in Table 1. All patients had been enrolled on an institutional review board approved protocol and treated at The University of Texas MD Anderson Cancer Center. According to the protocol, the 4D CT datasets for each patient had been acquired in 2.5-mm slices using a multislice CT scanner (Discovery ST, General Electric Healthcare Systems, Waukesha, WI) and the Real-Time Position Management (RPM) respiratory gating system (Varian Medical Systems, Palo Alto, CA). Ten CT datasets corresponding to the 10 phases in each equally divided respiration cycle (from the 0% phase, referred as the T0 CT, to the 90% phase, referred as the T90 CT) were reconstructed [20] for each 4D dataset. The end-of-exhalation phase CT (i.e., T50 CT) dataset from the 4D dataset acquired during simulation was selected as the reference and planning CT set. The TV was defined as the planning treatment volume (PTV) on the reference CT (i.e., PTV50), which was generated by defining the gross tumor volume (GTV) on the reference CT and then expanding the GTV by 8 mm to obtain the clinical TV (CTV) on the reference CT (i.e., CTV50) and by another 8-mm to obtain the PTV50. The MTV was defined as the PTV that was generated by a 16-mm expansion of the combined volume of the GTVs at all 10 phases, named the internal GTV (IGTV), i.e., 8-mm expansion of the IGTV to obtain the ITV and another 8-mm expansion of the ITV to obtain the PTV. The margins to expand from IGTV to ITV and ITV to PTV are currently adopted as the standard for the thoracic patients in our institution [21]. The prescription specified that the 4D dose (i.e., D_0_) of 63 Gy covers at least 95% of the PTV50 on the reference CT.

**Table 1:** Characteristics of the three patients used in the study.

Patient	**GTV50 (cm**^**3**^**)**	**PTV (cm**^**3**^**)**	Center-to-center tumor motion (cm)	Primary tumor motion direction	Prescribed dose (fxs × Gy/fx)
1	61.14	481.15	2.65	S-I	35 × 1.8
2	156.9	878.7	1.53	S-I	35 × 1.8
3	218.108	1204.06	3.42	S-I	35 × 1.8

Abbreviations: fx(s) = fraction(s)

The t4Dplan method was used to design the treatment plans for all patients. The non-uniform AppD for the PTV was derived from equations (3), (5), and (6), and the deliverable AppD was achieved by optimization using equation (7). The clinical treatment plan for all patients had been designed by our experienced dosimetrists based on the commonly used ITV method (i.e., the IMRT plan was designed to have uniform dose distribution to the PTV and minimal dose to normal tissues.). In this study, we re-optimized those plans and found that those plans could not be improved upon to our best effort and knowledge. To compare the plan based on the t4Dplan method with the plan based on the ITV method, the dose volume histograms (DVHs) for the PTV50 and normal structures (i.e., total lung, stomach, liver, spinal cord, and heart) were calculated based on deliverable AppD and 4D dose distribution. To assess plan quality with respect to target dose, we computed the conformity number (CN) for the PTV50 using the following definition [22]:(8)

where *TV*_*Dp *_is the TV covered by the prescribed dose and *V*_*Dp *_is the total volume enclosed by the prescription isodose surface. The CN ranges from 0 to 1, and the greater the CN, the better the prescribed dose's conformation to the target. A small CN indicates that either the target is not well covered by the prescribed dose (the first fraction of the equation) or the total volume of tissue receiving the prescribed dose was very large compared to the target (the second fraction of the equation).

The ratio (*R*_*prescribed_dose*_) of the prescribed dose volume in 4D dose distribution (*V*_4__*D*_) and deliverable AppD (*V*_*AppD*_) was calculated for plans derived from both the t4Dplan method and the ITV method for each patient to show the dosimetric effects of respiration-induced organ motion:(9)

### 2.3. Robustness of the t4Dplan method against interfractional variation of the respiratory pattern

**Figure 3:**
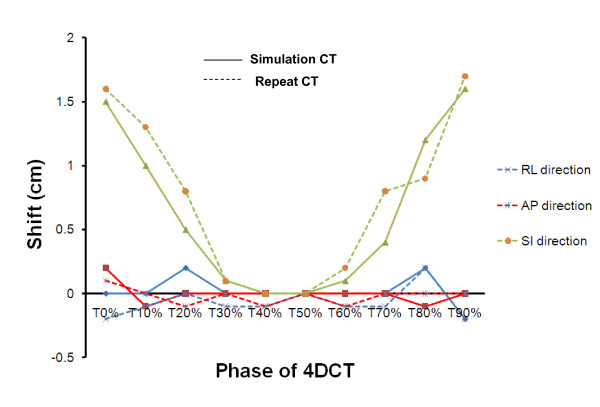
**The GTV motion on the simulation CT datasets (solid line) and repeat CT datasets (dashed line) for 10 phases of the respiratory cycle relative to the T50 phase in the right-left (RL) direction (blue color), anterior-posterior (AP) direction (red color), and superior-inferior (SI) direction (green color) for patient 2**.

**Figure 4:**
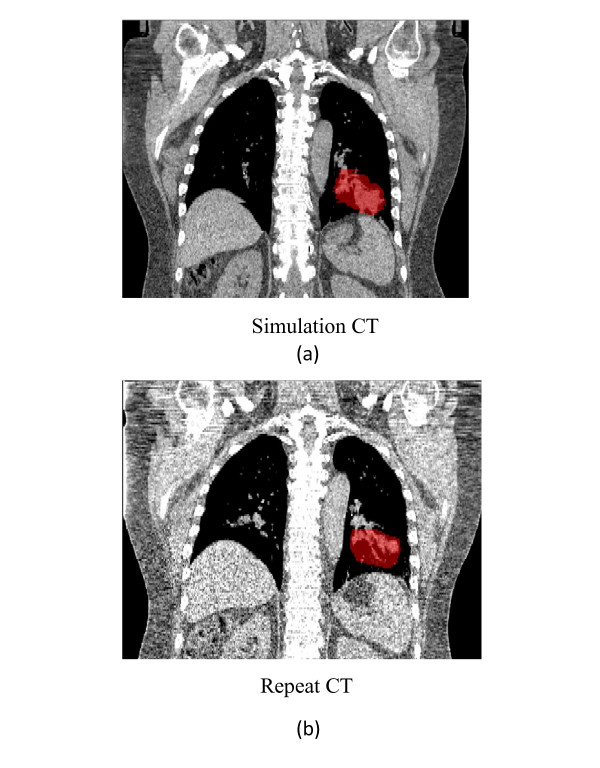
**Changes of GTVs (red color-wash) and other anatomic structures on coronal view of (a) simulation CT datasets and (b) repeat CT datasets for patient 2**.

To evaluate the robustness of t4Dplan method against the irregular breathing motion pattern, patient 2 and 3 were used. Since for these patients, not only 4D-CT were obtained during simulation to allow consideration of tumor motion in planning, but also several repeat4D CTs were obtained to assess the intra- and inter-fractional movement of the target volumes and the normal structures. One repeat 4D CT datasets acquired during week 2 of treatment for patient 2 and week 3 for patient 3 were selected to evaluate the robustness of the t4Dplan against inter-fractional variation in the respiratory pattern. Figure 3 shows the right-left (RL), anterior-posterior (AP), and SI shifts of the GTV on each phase (i.e., T0, T10, ...) relative to the T50 phase for both the simulation CT and repeat CT for patient 2. The repeat 4D CTs were also registered to the simulation 4D dataset using bony structures, and figure 4 shows the anatomic changes between coronal CT images obtained at simulation and week 2 for patient 2. Both figures 3 and 4 demonstrate the irregularity of breathing motion during fractional radiation treatments.

To evaluate whether the non-uniform dose prescription derived solely on the 4D simulation CT could still provide good target coverage and normal tissue sparing if the breathing pattern was irregular during fractional treatments, we recalculated the AppD in each phase of the repeat 4D datasets based on the plans designed using simulation CT and bony registration. The 4D dose distribution was cumulated and displayed on the reference CT (T50) of the repeat CT. The DVHs for the PTV50 and normal structures were calculated based on the 4D dose distributions to show the effects of inter-fractional variation in the respiratory pattern of the patient in the t4Dplan method.

## 3. Results

### 3.1. Theoretic and deliverable AppD

**Figure 5:**
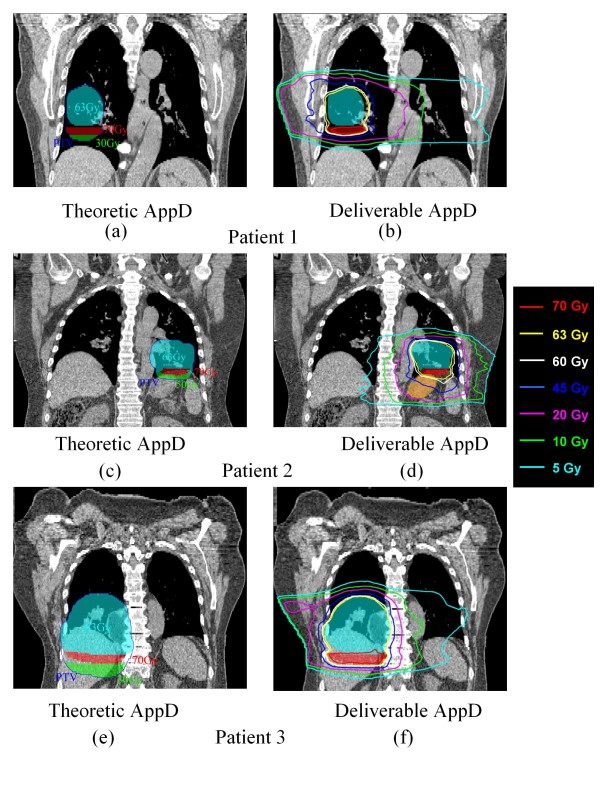
**The theoretic and deliverable AppDs for patient 1 shown on panel (a) and panel (b), for patient 2, shown on panel (c) and panel (d) and for patient 3, shown on panel (e) and panel (f) respectively**. The red and green color-wash on panel (a), (c) and (e) represent hot region (i.e. 70 Gy) and cold region (i.e. 30 Gy for patient 1 and 3, 50 Gy for patient 2) respectively for theoretic AppD. The blue color-wash on all the panels represents PTV50. The orange color-wash on (d) represents the stomach for patient 2.

Figure 5 shows the theoretic and deliverable AppD for patient 1 (figures 5(a) and 5(b)), patient 2 (figures 5(c) and 5(d)) and patient 3 (figures 5(e) and 5(f)). The theoretic AppD hot regions (i.e., 70 Gy, red color-wash on figure 5(a), (c) and 5(e)) for the PTV were located inferior to the PTV50 for all patients. Tumor motion in SI direction was 2.6 cm for patient 1, 1.5 cm for patient 2 and 3.31 cm for patient 3; motion in AP direction was 0.2 cm for patient 1 and 2, 0.85 cm for patient 3; and motion in RL direction was 0.5 cm for patient 1, 0.2 cm for patient 2. The SI direction was the dominant direction of tumor motion for all patients, which resulted in the hot regions of theoretic AppD appearing along the SI direction.

The optimized deliverable AppD was similar to the theoretic AppD for all patients, with the hot regions (i.e. 70 Gy isodose line in figure 5(b), figure 5(d) and figure 5(f)) mainly located inferior to the PTV50. The more similar the deliverable AppD was to the theoretic AppD, the more uniform the 4D dose distribution was in the PTV50 on the reference CT.

### 3.2. Normal-structure sparing in the t4Dplan method

Table 2 lists all the dosimetric indices for the 4D dose distributions calculated using the t4Dplan method and ITV method for all three patients. Since the tumor located in the middle lobe of the right lung for patient 1, the total lung sparing was significant using t4Dplan method compared to ITV method. For other two patients, as tumor located in the lower lobe of lung near the diaphragm and close to stomach (patient 2) or liver (patient 3), significant dose reduction for stomach and liver were observed using t4Dplan method compared to ITV method, respectively. And the total lung sparing was comparable using two methods. The reduction of the mean dose of heart of 4D dose distributions for all the three patients using t4Dplan method, were 3 Gy, 0.4 Gy and 0.2 Gy, respectively, from that using ITV method. The maximum cord dose of 4D dose distributions for patient 1 was slightly higher using t4Dplan method than that using ITV method, but far lower than cord tolerate dose (i.e. 45 Gy).

**Table 2:** Dosimetric indices of normal structures and PTV50 for 4D dose distributions calculated using the t4Dplan method and the ITV method for the three patients.

	Parameters	Patient 1		Patient 2		Patient 3	
		t4DPlan	ITV	t4DPlan	ITV	t4Dplan	ITV
**Total lung**	V5 (%)	55.4	63.9	32.2	32.7	41.0	42.5
	V10 (%)	30.2	35.7	24.2	25.4	25.0	26.2
	V20 (%)	19.1	24.3	20.7	21.7	17.3	18.2
	V30 (%)	14.8	19.8	18.3	17.6	13.0	13.4
	Mean (Gy)	13.2	16.3	12.8	12.9	11.3	11.8
**Spinal cord**	Max (Gy)	30.1	26.3	31.6	32.3	34.0	38.0
**Heart**	Mean (Gy)	10.8	13.4	23.8	24.2	18.8	19.0
**Stomach**	V40 (%)			8.2	21.4		
	V50 (%)			2.6	10.6		
	Max (Gy)			54.1	62.3		
	Mean(Gy)			19.3	27.3		
**Liver**	V30(%)					28.3	42.1
	Mean (Gy)					22.8	31.3
**PTV50**	V63 (%)	98	99.3	96.4	98.0	95.1	97.0
	CN	0.74	0.57	0.91	0.78	0.81	0.67
	R_63_	0.94	0.99	0.94	0.97	0.91	0.94

Abbreviations: R_63 _= ratio of volume greater than or equal to 63 Gy of 4D dose distribution to AppD; CN = conformity number; Vx = the volume of structures received dose >x Gy; Max = Maximum.

The PTV50 coverage by the 63 Gy prescribed dose (V63) for 4D dose distribution using the t4Dplan method was inferior to the coverage obtained using the ITV method from table 2. However, the CN for all patients using the t4Dplan method was significantly better than the CN of the ITV method. It indicates that the ITV method overestimated the TV. In other words, the ITV method overestimates the dose effect of respiration-induced target motion. Consequently, a large volume of normal tissue will be unnecessarily irradiated if the ITV method is used.

**Figure 6:**
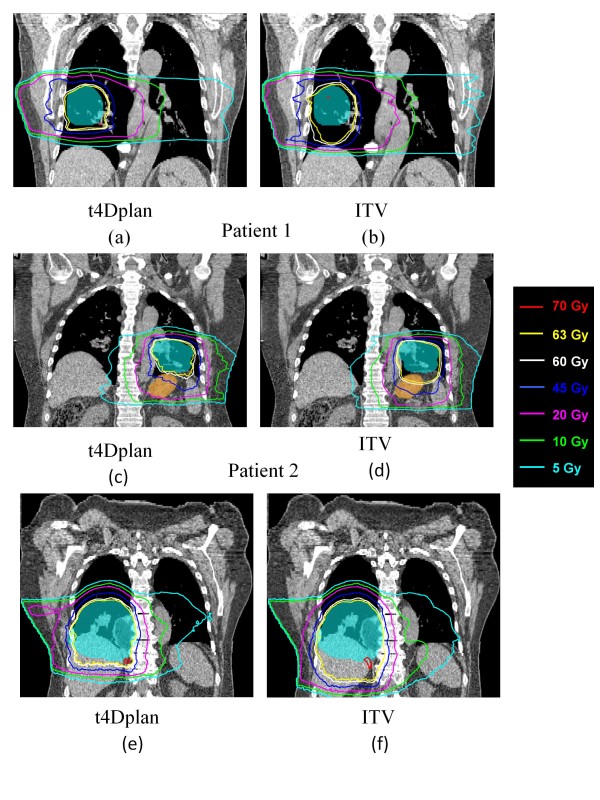
**4D dose distributions calculated using the t4Dplan method for (a) patient 1, (c) patient 2 and (e) patient 3, and the ITV method for (b) patient 1, (d) patient 2 and (f) patient 3**. The blue color-wash represents the PTV50. The orange color-wash represents stomach.

Figure 6 shows the 4D dose distributions calculated using the t4Dplan method and ITV method for the three patients respectively. The high-dose isodose lines (such as 63 Gy and 60 Gy) spread out especially in inferior direction for ITV method and conformed to the target very well for t4Dplan method. The low-dose isodose line pushed out slightly from target for ITV method compared to t4Dpaln method. It illustrates more normal tissue sparing using t4Dplan method than ITV method.

The R_63 _which is the ratio of volume greater than or equal to 63 Gy (prescription) of 4D cumulative dose distribution to apparent dose distribution is listed in table 2. They were all less than 1, meaning that target motion effectively smears the dose and reduces the high-dose volume. Comparing deliverable AppD and 4D dose distributions (i.e., figure 5(b) vs figure 6(a) for patient 1, figure 5(d) vs figure 6(c) for patient 2 and figure 5(f) vs figure 6(e)), it shows the prescribed isodose line (i.e., 63 Gy) on the 4D dose distribution was pushed in the superior direction and that it conformed well to the PTV50 (figures 6(a), (c) and 6(e)), since the dominant motion of the target was in the SI direction; hot regions (i.e., ≥70 Gy) were smeared out and significantly smaller on 4D dose distribution than that on the deliverable AppD. On the contrary, ITV method overestimated the target motion and the prescribed isodose line in ITV method enclosed many healthy lung tissues (figure 6(b), (d) and 6(f)). This fact is also reflected in CN index.

Since respiratory motion effectively reduces the volume receiving a high dose, as mentioned above, it may cause under dosing in the target if the plan is not designed to compensate for the motion-induced effects. Conversely, respiratory motion will create a more uniform target dose than designed if the plan is designed to compensate for the motion-induced effects.

### 3.3. Robustness of the t4Dplan method

**Figure 7:**
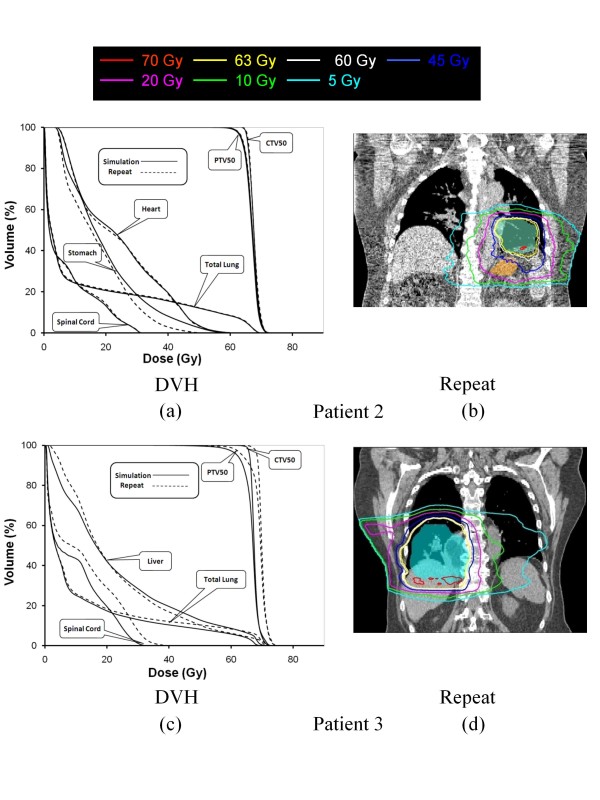
**(a) DVHs from 4D dose distribution calculated using the t4Dplan method on the simulation CT (solid line) and repeat CT (dashed line) for (a) patient 2 and (c) patient 3;4D dose distribution images calculated using the t4Dplan method on the repeat CT for (b) patient 2 and (d) patient 3**. The blue color-wash represents the PTV50. The orange color-wash represents stomach.

Figure 7 shows the DVHs and 4D dose distributions calculated on the simulation CT and repeat CT datasets for patient 2 and 3 (there was no 4D repeat CT available for patient 1). The coverage of CTV50 and PTV50 on the repeat CT was as good as that on the simulation CT for patient 2 and a little better than that on the simulation CT for patient 3. The DVHs of the normal structures were similar between the two distributions from simulation CT and repeat CT for both patients. This result indicates that there were essentially no significant changes in dose distribution for the plan designed using the t4Dplan even if there are some irregularities of respiration pattern for the patient from week to week. The stomach received fewer doses during week 2 of the treatment because volume of stomach was reduced during week 2 for patient 2. In other words, the t4Dplan method is robust against inter-fractional variation in the respiratory pattern.

### 3.4. Planning time for t4Dplan

Currently, the t4Dplan was implemented as a plug-in to Pinnacle (8.1x), which runs on an AMD Opteron 8220 CPU operating at 2800 MHz with an i387-compatible floating-point operation processor. It took less than 10 minutes to generate the DIR, and another 5 minutes to generate the non-uniform prescribed dose distribution. After the plan was optimized, it took 3 minutes to generate the 4D dose from the apparent dose.

## 4. Discussion

Our findings suggest that the t4Dplan method is an effective means of treatment planning, with features that make it superior to the ITV method, which currently is the most common strategy implemented clinically to compensate for respiration-induced target motion. Essentially, the t4Dplan method uses a smaller PTV while designing a heterogeneous target dose distribution for the planning CT. Because the t4Dplan method accounts for the effects of respiratory motion by adjusting dose within the target, the margin can be reduced relative to that in the ITV method plan, leading to more normal structures sparing. The rationale for the t4Dplan technique is as follows: 4D CT shows that all phases from T30 to T70 are usually similar to the T50 phase, which indicates that patients spent more time in the end-exhalation phase than in any other, that is, the tumor stays around the T50 position for a long period, while it remains at other positions, such as T0, for only short periods. The ITV method envelopes the tumor location in 10 phases to generate the treatment target, which means it weights the time that the tumor is in its T0 position the same as the time it is near its T50 position. The consequence is that the planned dose to the tumor's location around the T0 phase may be delivered to normal structures most of the time since the target moved out of the planned position most of time. The strategy of the t4Dplan method is to deliver a smaller dose to the tumor when the tumor is at its T0 position and then deliver higher doses to the tumor when it is close to its T50 position, which will compensate for the underdosing at the T0 position. In this way, normal structure sparing is maximized as the free-breathing patient undergoes radiotherapy.

Methods of designing treatment plans with non-uniform dose distributions to achieve better normal tissue sparing have been tested by several groups. For example, Li *et al *[23] used a simplified 4D dose calculation method to design a treatment plan with non-uniform dose. This method will only provide a treatment plan with non-uniform dose distribution. The simplified 4D dose calculation method used by Li *et al *(2006) directly convolves the 3D dose distribution with a probability distribution of the tumor over the breathing cycle and therefore does not accurately reflect the effects of breathing motion on the dose distribution. In t4Dplan method, the non-uniform target dose in the planning CT dataset is the apparent dose in the target, while the 4D dose is essentially uniform. The final result presented to the radiation oncologist yields a uniform dose distribution, and the plan is easily adopted by most practitioners. As the current report shows, the t4Dplan method can be readily implemented in the treatment planning system. We expect that this method will be readily adopted in centers where 4D CT scanners and related treatment planning systems are already available.

Other 4D planning approaches which designed the plans on mid-ventilation, mid-position scan or maximum- and minimum-intensity projection to account for the organ motion have been extensively studied by Wolthaus [24], Cuijipers [25] and Guckenberger [26]. According to their studies, a good dose coverage was still obtained even if the tumor was only fully within the prescribed iso-dose line during a small part of the breathing cycle. Therefore, a better normal tissue sparing was achieved compared to the ITV approach that overestimated the margins necessary for the breath motion. The t4Dplan which designs the non-uniform dose agrees with those previous studies. Cuijipers *et al *(2010) proposed to use a dosimetric margin, 80% iso-dose line of the prescribed dose which fully covers the PTV, to reduce margin compared to ITV approach. The coverage of 80% dose to the PTV for the three patients using t4Dplan was 91%, 96% and 90% respectively. Considering the fact that our t4Dplan is a motion adapted plan, in which the instantaneous hot and cold spots in the dose distribution delivered during various phases of the target motion are specially designed to compensate each other, the dosimetric margin derived using our approach is even smaller than that proposed by Cuijipers. In this sense, the t4Dplan is a further improvement. One major difference between ours and other works is that, although PTV is underdosed in the apparent dose distribution, PTV50 is adequately covered in 4D dose. Evaluating the coverage of PTV50 and normal tissue sparing on 4D dose distribution is much more easily accepted by physicians and a natural transition from ITV approach to the t4Dplan approach.

Our t4Dplan method essentially converts the 4D planning problem to a dose-painting problem based on 4D anatomic information. Currently, the dose-painting problem is a subject of intense research in the radiotherapy community [27-32]. The robustness of plans designed by dose-painting algorithms is widely recognized as a major challenge to advancing the technique [33]. In our study, we recalculated the plan designed using the simulation 4D CT by using a repeat 4D CT that exhibited the irregularity of breathing motion to test the robustness of our method. We found that our method is relatively robust against the irregularity of breathing motion for the two patients. One explanation is that our method did not require "exact" dose painting of the voxelized dose prescription. As shown in figure 5(a), (c) and 5(e), as long as the hot regions were "painted," the underdose due to the smaller margin would be compensated for. The other explanation was pointed by Cuijipers *et al. *(2010). There are two uncertainties affecting the robustness of any 4D planning method: 1) the inter-fractional variability of tumor motion due to the changes of the tumor motion in the breathing pattern of the patient and 2) and changes in the mean tumor position and tumor volume during the course of the treatment. As realized by Cuijipers *et al. *(2010) for breathing amplitude of 15 mm, a 30% change in amplitude corresponds to a change in a breathing margin of about 1 mm, implying that the impact of the first uncertainties is expected to be small. The second uncertainty is also small for most patients [25] and is also not considered in the PTV/ITV approach.

Other techniques, such as respiratory gating and breath hold, also showed critical organ sparing compared to ITV approach [34,35]. But for those patients who could not comply with the breath hold technique, our t4Dplan method and respiratory gating may be the good candidates to treat. Compared to the gating method, the t4Dplan does not require 4D delivery, instead, delivers the dose continuously and saves a lot of beam time, with the price of increasing around 15 minutes in the planning time. Considering the current treatment planning which required many rounds of trial and error, the increase in planning time is almost negligible. Although we demonstrated that our method was relatively robust using two patients as an example, in the clinical practice adopting our method, a motion monitoring protocol and adaptive planning strategy are suggested to ensure the target was adequately covered. For example, the 4D repeat CT should be taken and the robustness of the plan should be checked using the same approach for patients 2 and 3. If the target was found not adequately to be covered due to irregular breathing pattern, the new adaptive plan should be designed based on the new repeat 4D CT.

Of note, the three patient datasets used in this study illustrate the t4Dplan method as well as demonstrate the effectiveness and robustness of t4Dplan method. A study of applying this method to a cohort of patients is undergoing and summary of the study will be presented in near future.

## 5. Conclusions

The t4Dplan method is an effective and practical method for designing 4D treatment plans for tumors subject to respiratory motion. The t4Dplan method creates plans that permit better sparing of the normal structures than the commonly used ITV method, which overcompensates for the dosimetric effects of respiration-induced motion to the target. The t4Dplan method does not require 4D treatment delivery and therefore can be readily adopted in centers where 4D CT scanning is already available.

## Competing interests

The authors declare that they have no competing interests.

## Authors' contributions

All authors read and approved the final manuscript. XZ originated the idea, XL and XW carried out all the CT evaluation, target delineation. XL also drafted the manuscript. XL and YL contributed to the acquisition of the data and the plan optimization. XW and XZ contributed to the final version of the manuscript.
